# The Intrinsic Resolution Limit in the Atomic Force Microscope: Implications for Heights of Nano-Scale Features

**DOI:** 10.1371/journal.pone.0023821

**Published:** 2011-08-30

**Authors:** Sergio Santos, Victor Barcons, Hugo K. Christenson, Josep Font, Neil H. Thomson

**Affiliations:** 1 School of Physics and Astronomy, University of Leeds, Leeds, United Kingdom; 2 Departament de Disseny i Programació de Sistemes Electrònics, UPC - Universitat Politècnica de Catalunya, Manresa, Spain; 3 Department of Oral Biology, Leeds Dental Institute, University of Leeds, Leeds, United Kingdom; University of Nebraska-Lincoln, United States of America

## Abstract

**Background:**

Accurate mechanical characterization by the atomic force microscope at the highest spatial resolution requires that topography is deconvoluted from indentation. The measured height of nanoscale features in the atomic force microscope (AFM) is almost always smaller than the true value, which is often explained away as sample deformation, the formation of salt deposits and/or dehydration. We show that the real height of nano-objects cannot be obtained directly: a result arising as a consequence of the local probe-sample geometry.

**Methods and Findings:**

We have modeled the tip-surface-sample interaction as the sum of the interaction between the tip and the surface and the tip and the sample. We find that the dynamics of the AFM cannot differentiate between differences in force resulting from 1) the chemical and/or mechanical characteristics of the surface or 2) a step in topography due to the size of the sample; once the size of a feature becomes smaller than the effective area of interaction between the AFM tip and sample, the measured height is compromised. This general result is a major contributor to loss of height and can amount to up to ∼90% for nanoscale features. In particular, these very large values in height loss may occur even when there is no sample deformation, and, more generally, height loss does not correlate with sample deformation. DNA and IgG antibodies have been used as model samples where experimental height measurements are shown to closely match the predicted phenomena.

**Conclusions:**

Being able to measure the true height of single nanoscale features is paramount in many nanotechnology applications since phenomena and properties in the nanoscale critically depend on dimensions. Our approach allows accurate predictions for the true height of nanoscale objects and will lead to reliable mechanical characterization at the highest spatial resolution.

## Introduction

The AFM is a powerful surface characterization tool allowing the height and the width of nanoscale features to be measured routinely with nanometer and sub-nanometer resolution [1], [2], [3], [4], [5]. Recent advances in the field are allowing researchers to investigate [6] and identify [7], [8] the chemical structure of single molecules and nanoscale crystals. In dynamic imaging modes (dAFM) [9], [10], the excitation of higher harmonics [11], [12] and the relationship between the fundamental frequency and higher modes [13], [14], [15] hold promise for the determination and simultaneous acquisition of mechanical and chemical maps at nanometer length scales.

**Figure 1:**
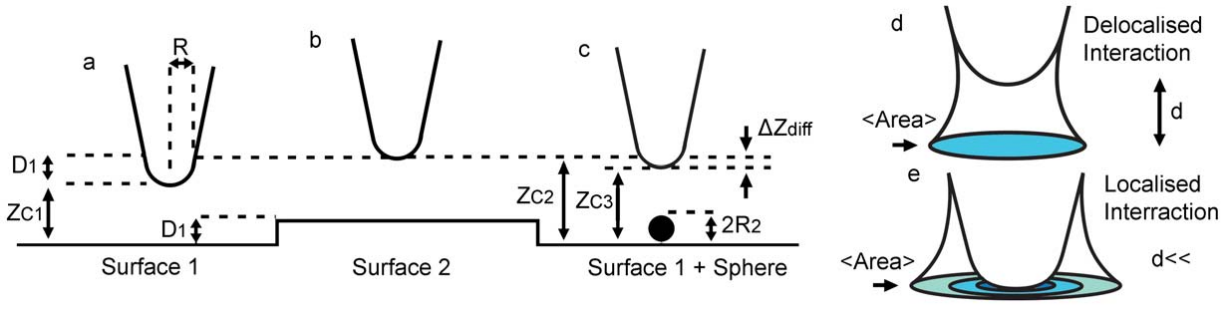
Scheme of the interaction between the tip an infinite surface and a finite sample feature. (a), Scheme of the interaction between a tip and a flat surface (surface 1). The equilibrium cantilever-surface separation (z_c_) is defined as z_c1_. (b) When the tip is over a second surface (surface 2) of height D_1_ the z-piezo actuator increases z_c_ to z_c2_. The displacement z_c2_−z_c1_ is termed the apparent height of the surface. (c) When encountering a sphere of height 2R_2_ the z-piezo varies z_c_ to z_c3_. Scheme of the interaction area between the end of the AFM tip and a surface at (d) large and (e) close distances. The force is not localized at a single point but acts over a finite area: <Area>.

**Figure 2:**
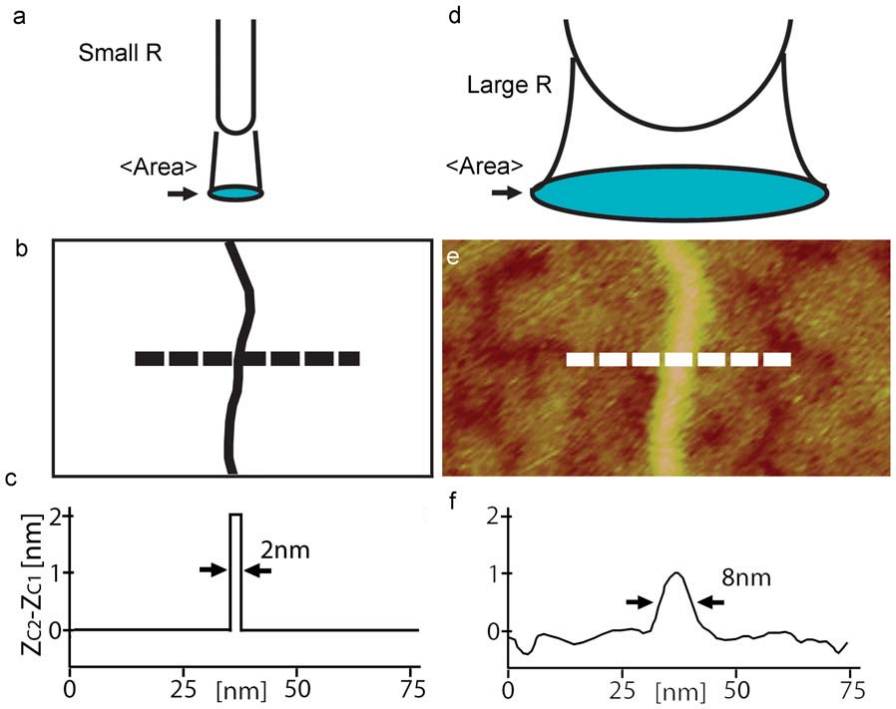
Scheme of a hypothetical point area versus experimental outcomes where the area is finite. (a) Scheme of the small <Area> (blue) with a hypothetical delta-function tip on a perfectly flat surface. (b) Hypothetical topographic scan of a dsDNA molecule with such a tip and surface. (c) Scheme of the perfect height profile (dashed line) achieved with this hypothetical set-up. (d) Scheme of the larger <Area> (blue) resulting from a common physical tip (e.g. 5<R<30 nm). (e) Topographic scan of a dsDNA molecule on mica with a standard tip in AM AFM in air. (f) Experimental height profile (dashed line). The apparent height is approximately 1 nm or half its true height and it appears lower and broader. Breaks in the layer of surface contamination allow the apparent height of the DNA to be measured relative to the mica surface. The surface layer with troughs and valleys accounts for approximately 0.2 nm. Experimental parameters: (e) A_0_ = 3 nm and A_sp_/A_0_ = 0.9 (normalized set-point).

Still, there is a fundamental problem concerned with the 3D information that is obtained at very short length scales. Typical measurements of nanoscale features with an AFM give an apparent height that is almost always lower than their known true height [10], [16], even when feedback gains are optimized. In particular, the apparent height of dsDNA as measured in AFM can be anything from 10 to 90% [16], [17], [18], [19], [20], that of its true height even after careful calibration of the instrument; the nominal true diameter of B-form dsDNA should be 2 nm [21] according to X-ray measurements [22]. Some have reported that changes in elastic modulus of the sample and/or the attractive component of the force [23] can produce variations in the cantilever-surface separation (z_c_) leading to loss of true height [23], [24], [25] (see Fig. S2 in the supplementary for details). Others have concluded that contamination or salt deposits around molecules on typical support surfaces for molecules, such as mica [26], and/or dehydration could be partly responsible for height reduction [16]. Generally, it has been commonplace to attribute height loss to sample deformation [16], [27], [28] and/or high forces[10], [13], [27], [28], [29], whenever it is observed. Here we show that the finite size of the surface feature (e.g. the sample) and the tip radius (R) are intrinsically responsible for the loss of true height in all types of AFM. This is a direct consequence of the fact that the force comes from an effective area of interaction <Area> (Figs. 1, 2) which is larger than a single point directly under the tip. Our results show that there is a resolution limit in the atomic force microscope, which not only affects the lateral resolution, but also affects height measurements of nanoscale sample features. In essence, the integrated force between the tip and the sample is spread-out laterally in an effective area of interaction with a certain pressure distribution. Thus, when the feature to be measured becomes smaller than this effective area of interaction, the height measured by the AFM, in any mode, is a convolution between the height of the surface feature and the height of the supporting surface. We demonstrate this fundamental limit using AM AFM, but our approach has the potential to be generalized to include all forms of probe microscopy where <Area> is finite. Comparison of AM AFM experimental data on single isolated DNA and protein molecules with a model which includes the tip-sample interaction area, leads to quantification of intrinsic height loss in the different imaging modes (non-contact (NC) and intermittent contact (IC)).

## Results and Discussion

The origins of apparent height in AFM can be understood qualitatively by observing that, when topography occurs, the tip-sample forces occurring are fundamentally different from the forces occurring when the tip interacts with an infinite and flat surface from a geometrical point of view (see Fig. S1 in the supplementary for details). For example, in Fig. 1a, initially, the surface forces originate from the interaction between the tip radius, R and an infinite surface (surface 1); then a given cantilever-surface separation z_c1_ follows. Over a second surface 2 with the same properties as surface 1, and with true height D_1_ relative to the level surface 1, z_c_ is now z_c2_ (Fig. 1b). Since the surface properties and local geometry between surface 1 and 2 have not changed, the cantilever dynamics remain the same over both surfaces. Thus, it follows that z_c2_−z_c1_ = D_1_ and thus, the true topography is measured. Now, let us assume that the tip encounters a small feature such as a sphere of radius R_2_ on surface 1 with the same material properties as surfaces 1 and 2 (Fig. 1c). In this case, the tip-surface forces include not those from the tip radius R and surface 1 alone, but those from surface 1, tip radius R and a sphere with true height 2R_2_. In practice, this is particularly relevant when <Area> is bigger than 2R_2_, where 2R_2_ is the diameter or size of the nanoscale feature. In order to obtain the true height of the sphere the separation should now be z_c3_ = z_c1_+2R_2_ and if 2R_2_ = D_1_, z_c3_ = z_c2_ would be required. This would only happen if the force for the tip-surface-sphere system was coincidently equivalent to that between the tip and the infinite surface 2. In practice a difference in separation, or z-piezo motion Δz_diff_, follows. This difference is in fact Δz_diff_ = z_c2_−z_c3_ where z_c3_−z_c1_ is the apparent height. It is significant that we do not refer here to changes in the magnitudes of the forces due to the chemical or material properties of the samples, but to changes based purely on geometry. A comparison between the effects of a hypothetical point-like area of interaction (Figs. 2a–b) and the experimental or real effects for a dsDNA molecule on a mica surface (Figs. 2c–d) is shown in Fig. 2. A tip of finite size means that the height profiles broaden and become lower in the experimental case. In essence, the height information is spread out across the width of the interaction area.

The fact that the interaction occurs in a finite area where the tip-sample force adds to the tip-surface force can be demonstrated with the use of simulations. Here, we use a standard model (1–2) based on a point mass and a spring [30], [31] where the tip-sample forces (3–4) are added. Note that the sample here is modeled as a sphere of radius R_2_. The van der Waals [32] and the Derjaguin Muller Toporov (DMT) [33] contact forces for a tip and an infinite surface are used to model the forces. The long range van der Waals forces prevail in any AFM experiment since they are the consequence of the ever present electromagnetic field fluctuations [10] in vacuum, ambient and liquid environments [32]. The short range repulsive forces arise from the quantum mechanical Pauli exclusion principle that impedes matter interpenetration. Nevertheless, it is typical [10], [34] in AFM to model short range repulsive forces with the use of contact mechanics models where the deformation of the surface, as load is applied to it, is responsible for this repulsive reaction force. Thus, these are the forces that are prevalent in any AFM experiment and dominate in air in situations where capillary neck formation is absent. We distinguish between a “surface” and a “sample”, where the sample is any nanoscale feature on the surface.
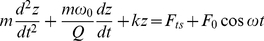
(1)


In (1) the effective mass of the AFM probe is defined [35] as m = k/(ω_0_)^2^. Here the spring constant of the cantilever is k, the natural angular frequency is ω_0_, the driving force is F_0_cosωt and F_ts_ is the net tip-sample force. Moreover, z is the instantaneous position of the tip and it is measured from the equilibrium position for the unperturbed cantilever.

For the tip-surface scenario we have,
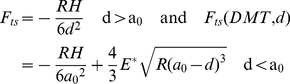
(2)


Here E^*^ is the effective elastic modulus as typically defined in contact mechanics [36], H is the Hamaker constant [32], R is the tip radius, a_0_ is an intermolecular distance introduced to avoid divergence [30] and d is the instantaneous distance between the tip and the surface. For the tip-sphere-surface interaction we have,
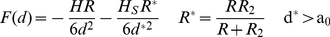
(3)and
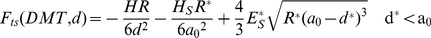
(4)


**Figure 3:**
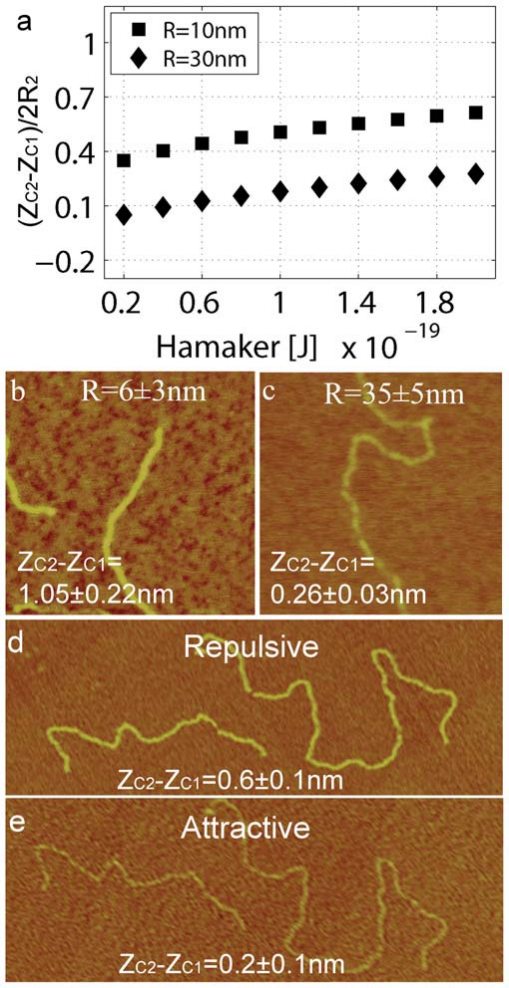
Model versus predictions of apparent height. (a) Model predictions obtained for the normalized apparent height (z_c2_−z_c1_)/2R_2_ as a function of (H_s_). The normalized apparent height (z_c2_−z_c1_)/2R_2_ increases with H and decreases with R. Significantly, (z_c2_−z_c1_)/2R_2_ can be smaller than 0.1 or 10% even though all the data has been obtained in the nc mode. In (b) and (c) experimental topographic images of dsDNA molecules on mica show the relationship between R and (z_c2_−z_c1_)/2R_2_. As in the simulations (z_c2_−z_c1_)/2R_2_ increases with decreasing R. In (d) and (e) a single molecule has been imaged in the repulsive and the attractive regimes and the higher value of (z_c2_−z_c1_)/2R_2_ has been obtained in the former. Simulation parameters: A_0_ = 1 nm, A_sp_/A_0_ = 0.95, E_s_ = 0.2 GPa, γ = 50 mJ/m^2^ (surface energy of the surface) and H = 10×10^−20^ J (Hamaker of the surface). Experimental parameters: (b–c) A_0_ = 4 nm and A_sp_/A_0_ = 0.9; (d) A_0_ = 20 nm and A_sp_/A_0_ = 0.9; (e) A_0_ = 2 nm and A_sp_/A_0_ = 0.9.

**Figure 4:**
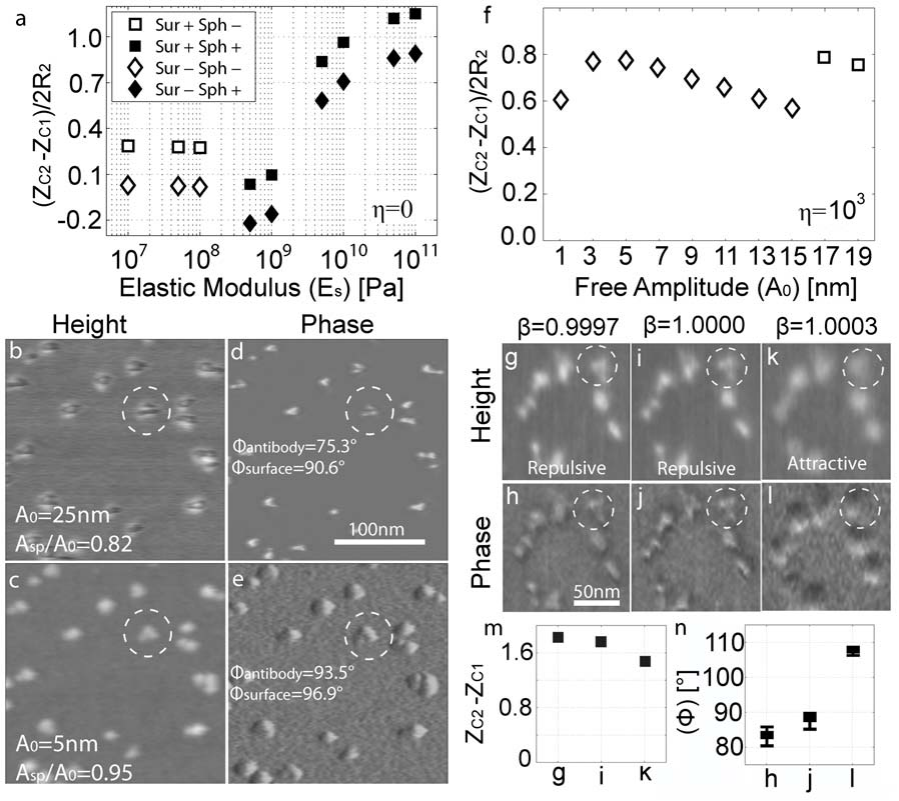
The effects of the elastic modulus and the free amplitude on apparent height. (a) Simulations of the tip-surface and tip-surface-sample interaction. Normalized apparent height (z_c2_−z_c1_)/2R_2_ versus E_s_. Note that (z_c2_−z_c1_)/2R_2_ might initially decrease with increasing E_s_. The attractive and repulsive regimes might be reached only on either the surface or the sample. The outcomes of the four possibilities are shown with squares and rhombuses where Sur and Sph stand for surface and sphere respectively. The minus and plus signs refer to the attractive and the repulsive regime. (b) Topography and (c), phase contrast of IgG antibodies on mica where height reversal is observed. The attractive regime is stably reached on the surface whereas the repulsive regime is immediately reached where any topography occurs (e.g. the antibodies). This phenomenon leads to the high contrast in phase compared to where the attractive regime (d–e) is reached both on surface and the antibodies. (f) Simulations of the tip-surface and tip-surface-sample interaction. An extra viscoelastic component (η) has been added to the contact region (see Figs. S3, S4, S5, S6 in the supplementary for details). Normalized apparent height (z_c2_−z_c1_)/2R_2_ versus A_0_ for A_sp_/A_0_ = 0.88. The attractive regime is reached both on the surface and the sphere for the lower values of A_0_ and (z_c2_−z_c1_)/2R_2_ initially increases and then decreases with A_0_. For larger values of A_0_ the repulsive regime is reached on the surface only and (z_c2_−z_c1_)/2R_2_ increases. (g–l) Topography and phase images of IgG antibodies with different normalized driving frequencies (β = f/f_r_) and constant A_0_ and A_sp_. Here (z_c2_−z_c1_)/2R_2_ increases in the repulsive regime. The respective values for height and phase are shown in (m–n). Simulation parameters: (a) A_0_ = 12 nm, A_sp_/A_0_ = 0.70, E = 1 GPa, γ = 50 mJ/m^2^ and H = 10×10^−20^ J; (f) A_sp_/A_0_ = 0.88, E = 10 GPa, E_s_ = 0.1 GPa η = 1000 and the rest as above. Experimental parameters: (b–e) as detailed in the figures; (g–l) A_0_ = 24 nm and A_sp_/A_0_ = 0.7.

Thus, the distance d is the typical instantaneous tip-surface distance in the absence of sample (2) whereas for the tip-sample forces (3–4) we use the parameter d^*^ as the effective distance between the tip and the “sample”. Then d^*^ is defined as the surface distance minus the height of the surface feature, which, in the case of a sphere of radius R_2_, gives d^*^ = d-2R_2_. Note that (3) is an approximation to the true sphere-sphere interaction. Nevertheless, it can be shown that this approximation is justified when results are compared to those obtained when using the true, more convoluted, expression (see section 4 and Figs. S10, S11, S12 in the supplementary). From this model, several conclusions follow. First, in the non-contact (nc) mode of operation, where mechanical contact never occurs, the interpretation of height reduction as tip-sample-deformation can be ignored by definition. Yet, we still get (z_c2_−z_c1_)/2R_2_<1 (Fig. 3a). Thus, other interpretations for height reduction should be sought. Second, a dependency on R is observed (Fig. 3a) that agrees with experimental observations (Fig. 3b–c). Third, the interpretation of increasing height (z_c2_−z_c1_) with decreasing forces is also ruled out. This is because it can be shown both experimentally (Fig. 3d–e) and with simulations (Fig. 4) that z_c2_−z_c1_ can increases with increasing force or free amplitude A_0_ and/or deformation (see Figs. S5, S6 in the supplementary for details). Fourth, simulations show that the apparent height might increase with decreasing elastic modulus of the sample (E_s_) (Fig. 4a). Fifth, typically two force regimes are distinguished and, in the literature [30], these are termed the attractive and the repulsive regimes according to whether the average force per cycle is attractive or repulsive. Furthermore, the size of the nanoscale feature directly affects the transition as a consequence of the geometry dependency of the forces. In short, the repulsive regime is more readily reached with decreasing sample size (not all data shown). Significantly, the model predicts that height reversal might occur when the attractive regime is reached on the surface and the repulsive regime is reached on the molecules (Fig. 4a). In Fig. 4 we write Sur for surface and Sph for the sphere sample. Then, we differentiate between reaching the attractive and the repulsive regimes with minus − and plus + signs respectively. For example Sur + and Sph + means that the attractive regime has been reached on both the surface and the sphere sample. An experimental example of height reversal for antibody molecules is shown in Figs. 4b–d. Subsequent scanning with lower values of A_0_ allows attractive imaging on both the surface and the antibodies (Figs. 4c and 4e). Importantly, molecular damage is not observed neither when height reversal occurs in the repulsive regime (Fig. 4c) nor in the attractive regime (Figs. 4d–e). Sixth, in the repulsive mode of operation, and according to simulations (Fig. 4f), the value z_c2_−z_c1_ might actually increase relative to the attractive regime. This can also be observed experimentally in Figs. 3d–e and Figs. 4g–n for DNA and protein samples respectively (see Fig. S9 in the supplementary file for details on the relationship between drive frequency, deformation and apparent height in the repulsive regime). Seventh, significantly and reiterating, height loss and deformation (δ) do not directly follow from each other, that is, in general δ/2R_2_≠(z_c2_−z_c1_)/2R_2_ (see Figs. S3, S4, S5, S6 in the supplementary for details on the model). This can be easily verified with the help of simulations (see Figs. S5, S6 in the supplementary for details). Moreover, experimental results further show that the apparent height of soft biomolecules can in fact increase with increasing free amplitude even in the repulsive regime (see Figs. S7, S8 in the supplementary for details). From this, it follows that observation of decreasing height with variations of the operational parameters does not involve that more sample deformation has been produced with one or the other set of operational parameters. Finally, it is worth mentioning some of the implications of the dependency of apparent height on tip radius R. First, note that since R typically changes from experiment to experiment, and even from scan to scan in the same experiment [19], [37], the apparent height of a surface feature is bound to change as a consequence of this dynamic character of the value of R. Nevertheless, we have recently shown that the effective value of the tip radius can be kept relatively constant in experiments [37]. In particular this can be done by submitting the tip to ever increasing interaction forces by slowly increasing the free amplitude prior to carrying out the experimental work. Secondly, differences in the way the tip is fabricated will also affect tip stability, wear and manufacturer's nominal values. Nevertheless, overall, our results predict that the apparent height of surface features will change from experiment to experiment as a consequence of changes in R due to lack of tip stability.

### Conclusions

In summary, we have shown that the geometry of the tip-surface-sample interaction area can induce deviations in the apparent relative to true height of up to 90%. The intrinsic resolution limit in the atomic force microscope causes height information to be spread-out laterally across the tip-sample interaction area resulting in loss of height for features of nanoscale lateral dimensions.

Since phenomena occurring in the nanoscale, where the properties of matter vary from those displayed by atoms or macro-objects, are largely dependent on the dimensions of the features, accurate understanding of measurements is paramount. In this respect, the results presented in this article are relevant to all fields of research using the AFM to characterize the dimensions of nanoscale samples. Our results have further shown that the effects of the size of the sample here described are particularly relevant in the nc mode of operation where very small and attractive forces are typically involved. Since this is a common mode of operation when imaging soft matter, and for preserving the sharpness of the tip, special care should be taken in these experiments to interpret data. We have further shown that even with relatively small forces, and as long as the effective area of interaction is larger than the lateral size of the feature, the true apparent height will never be obtained. For example, the forces in the non-contact mode in Fig. 3a are much smaller than those obtained in the repulsive regime in Fig. 4f when using the larger free amplitudes. Recall that the repulsive forces rapidly escalate with increasing free amplitude [30], [37].

More generally, predicted heights with this model and future adaptations can be compared with experimental data and used to deduce the real height values. This will therefore enable decoupling of intrinsic height loss from other sources thus allowing local mechanical properties of molecules and surfaces to be more rigorously defined at the nanoscale. This interpretation brings AFM into analogy with other forms of microscopy where there is an intrinsic resolution limit.

## Materials and Methods

The experimental data in this work has been obtained with a Nanoscope Multimode IIIa AFM (Veeco). Only cantilevers for which a Lorentzian response in air could be obtained have been used. Both dsDNA (1074 bp) and Immunoglobulin G (IgG) antibodies samples have been used as model systems where details on sample preparation can be found in the literature [38]. Furthermore, here some experimental and simulation parameters always take the same numerical values throughout for simplicity since emphasis is placed on the concepts discussed rather than on specific details. These are S = [Spring constant (k), Quality factor (Q), elastic modulus of the surface (E), elastic modulus of the tip (Et), Poisson's coefficient (ν), radius of the sphere or sample (R2), driving frequency (f0)] with S = [40 N/m, ∼500, ∼10 GPa [39], 120 GPa, 0.3, 1 nm, ∼300 kHz] throughout. For simplicity, we have also always worked at resonance except otherwise stated. The equation of motion (1) has been implemented in Matlab and Simulink [40] and solved with a standard fourth order Runge Kutta algorithm. The equations for the tip-surface interaction (2) have been used to obtain the cantilever-surface separation z_c1_ whereas the sample forces (3–4) have been used to obtain the cantilever-sample separation z_c2_. One can choose any set of operational parameters and cantilever-surface-sample properties to obtain a difference z_c2_−z_c1_. This is the predicted apparent height.

## Supporting Information

Figure S1 Scheme of the effective interaction area, <Area>, of a tip in the proximity of a surface and its relationship to sensitivity and proximity of surface features. The force per unit area might change from the point just under the tip (P_1_) to other points inside <Area> (e.g. P_2_). This is exemplified with differences in contrast where, in the example, the darker the colour the greater the localisation. For example the force per unit area (or localisation) is larger in (a) d_1_ than (b) d_2_ where d_2_>d_1_.
(TIF)

Figure S2 Simulations where the predicted apparent height of surface samples is shown. (a) Changes in apparent height (z_c2_−z_c1_) in the non-contact mode for a tip-surface-sample system (spheres, R_2_ = 1–4 nm) for several values of tip radius (R = 10, 20 and 30 nm). The value z_c2_−z_c1_/2R_2_ decreases with R_2_ and increases with decreasing R even though the characteristic parameters of both sample (i.e. a sphere) and surface are the same, except for the elastic modulus. However, since all measurements are in the nc mode the value of E is irrelevant here. The parameters are: A_0_ = 3 m, E_s_ = 0.2 GPa, E = 10 GPa, γ = 30 m (surface and sphere) J/m^2^, H = 6×10^−20^ J (surface and sphere), A_sp_/A_0_ = 0.90. Tip-surface systems. (b) Changes in apparent height (z_c2_−z_c1_) due to variations in the local value of H in the nc mode for R = 10 and 30 nm. The parameters are: A_0_ = 3 m, E = 0.2 GPa, and A_sp_/A_0_ = 0.90, γ = 30 m J/m^2^ (H = 6×10^−20^ J) and the rest as above. The reference value is H = 10×10^−20^ J (z_c1_). (c) Changes in apparent height due to variations in the local values of the E in the repulsive regime. Values for R = 10 nm and R = 30 nm overlap in this case because very large free amplitudes were used except for the case E = 10 MPa and R = 30 nm for which the repulsive regime could not be reached. The parameters are: A_0_ = 60 nm, A_sp_/A_0_ = 0.70 and the rest as above. The reference value is E = 1 GPa (z_c1_).
(TIF)

Figure S3 Predictions of apparent height as a function of elastic modulus of the sample including viscoelasticity. (a) As Fig. 4a, i.e. no viscoelasticity. (b) Consequences of including viscoelasticity (η = 1000 Pa•s^2^). The meaning of the markers is also the same as that in Fig. 4a. This viscoelastic term, previously used in the literature for the typical case of tip-surface only [34], [41], provides a dissipative mechanism in which dissipation increases with indentation. The height difference between squares and rhombuses is a consequence of the attractive regime being reached on the sample sphere in the former and the repulsive in the latter case.
(TIF)

Figure S4 Predictions for the normalised deformation (or indentation) for a sample sphere of R_2_ = 1 nm. The indentations correspond to those in Fig. 4a and S3. The maximum deformation occurs for intermediately compliant samples (e.g. 0.5<E_s_<2 GPa) both when no viscoelasticity is allowed (squares) and when it is present (rhombuses); less so in the latter. The legends are different to those in Figs. 4a and S3; S4 only shows whether the force regime is attractive (−) or repulsive (+) on the sphere (Sph).
(TIF)

Figure S5 Predictions of apparent height as a function of free amplitude, elastic modulus and Hamaker. Apparent height (z_c2_−z_c1_)/2R_2_ for (a) a compliant sample (E_s_ = 0.1 GPa) and (b) an intermediately compliant sample (E_s_ = 2 GPa) as a function of free amplitude for a constant set-point. Where the markers overlap these are shown explicitly with arrows pointing to the respective points. If no viscoelasticity is allowed total deformation is predicted in some cases (data not shown). The parameters are: A_sp_/A_0_ = 0.88, E = 10 GPa, γ = 60 m J/m^2^, H = 12×10^−20^ J and η = 1000 Pa•s^2^.
(TIF)

Figure S6 Predictions for the Normalised deformation as a function of A_0_ corresponding to the same parameters used to obtain Fig. S5. The deformation monotonically increases with A_0_ but does not correspond to a pattern that might be expected if reduction in apparent height, (z_c2_−z_c1_)/2R_2_ was a consequence of deformation only (c.f. Figs. S5, S6). In particular, it is remarkable that even though there is a step up in (z_c2_−z_c1_)/2R_2_ when the repulsive regime is reached on the surface (A_0_>15 nm) the deformation still increases.
(TIF)

Figure S7 Sequence of topographic scans of two 800 Kbp dsDNA molecules. (a–o) A_0_ has been systematically increased (1<A_0_<20 nm) while keeping the set-point high and driving at resonance. All scans have been acquired with A_sp_/A_0_∼0.9 except for (o) where A_sp_/A_0_ has been slightly reduced compared to (n), in order to allow comparison of apparent heights between the L-state (attractive regime) and H-state (repulsive regime) there. The value of R was approximately 10 nm.
(TIF)

Figure S8 Experimental values of (z_c2_−z_c1_)/2R_2_ and phase shift corresponding to Fig. S7. (a) Experimental values of (z_c2_−z_c1_)/2R_2_, where R_2_ = 1 nm has been taken as the reference value for the true radius of dsDNA molecules. Even though the DNA molecules are certainly not perfectly circular in cross-section, the predicted values can be taken as a first approximation to the phenomenon. Average values and error scales are shown. Note that an extra value is shown at the end for A_0_ = 4.5 nm. This corresponds to a control scan obtained to compare the values of (z_c2_−z_c1_)/2R_2_ after the sequence (scan not shown). The two values of A_0_ = 4.5 nm are coloured blue to allow easy comparison. There are also two values for A_0_ = 19.5 nm corresponding to the attractive and repulsive regions in Fig. S7o; shown in red. These allow for comparison between regimes for these larger values of A_0_ and, as predicted (Fig. S5) (z_c2_−z_c1_)/2R_2_ is larger in the repulsive regime! This is despite δ also being larger in the repulsive regime. This outcome is also demonstrated in the main text in Fig. 3. Nevertheless it is important to realise that, in general, these type of simulations predict that for relatively large values of A_0_, (z_c2_−z_c1_)/2R_2_ can be larger in the repulsive regime for a given A_sp_/A_0_. From this, it does not necessarily follow that (z_c2_−z_c1_)/2R_2_ increases with A_0_. In particular, the tendency for increasing A_0_ is that (z_c2_−z_c1_)/2R_2_ decreases with increasing A_0_ once in the repulsive regime and this is confirmed in simulations for A_0_>19 nm such as that shown in Fig. S5. Experimental evidence of this behaviour can be found in the literature [19]. (b) Corresponding phase shifts where L and H on top of each data point stand for L and H-states respectively.
(TIF)

Figure S9 Predictions relative to drive frequency in the repulsive regime. Simulations of (a) Normalized δ and (b) (z_c2_−z_c1_)/2R_2_ for E_s_ = 2 GPa (filled) and 5 GPa (outlined) of R_2_ = 1 (squares) and 2 nm (circles). A value of A_0_ = 24 nm and set-point of A_sp_/A = 0.80 has been used throughout. No viscoelastic term has been used (e.g. η = 0 Pa•s^2^). Here δ is seen to increase with increasing drive frequency while ((z_c2_−z_c1_)/2R_2_) decreases. All values have been obtained in the repulsive regime. There is a clear relationship between increasing δ and decreasing (z_c2_−z_c1_)/2R_2_. The parameters are: H = 10×10^−20^ J and R = 10 nm and the rest as indicated in the main text.
(TIF)

Figure S10 Comparison between exact and simplified van der Waals expressions for two spheres. Force versus distance between a tip (R = R_1_) and a sphere (R_2_) for (a) R = 10 nm and (b) 20 nm. The forces predicted by the simplified van der Waals equations are shown (filled) against the true values (outlined) for R_2_ = 1 (squares) and 5 nm (rhombuses) respectively.
(TIF)

Figure S11 Comparison between exact and simplified van der Waals expressions for an infinite surface and an sphere. Comparison between the simplified equations (filled) for the van der Waals force between a sphere of radius R and an infinite and flat surface and the true equations (outlined). The forces are shown as a function of distance d. The relationships are shown for R = 10 (squares) and 20 nm (rhombuses). The simplified form closely follows the true equation even for distances as large as 3 and 4 nm.
(TIF)

Figure S12 Comparison between the results for apparent height between the exact and simplified van der Waals expressions in the nc mode. Simulations showing (z_c2_−z_c1_)/2R_2_ for several values of R and R_2_. The true equations for the van der Waals interactions (filled) have been used against the simplified equations (outlined) for R = 10 and 30 nm respectively. The true values produce only slightly smaller values for the apparent height. All data was achieved in the non contact mode. The parameters are: A_0_ = 1 nm, A_sp_/A_0_ = 0.95, E_s_ = 5 GPa, E = 10 GPa, H = 6.1×10^−20^ J, γ = 30 m J/m^2^ and all other parameters as detailed in the main article.
(TIF)
