# Supplementary figures and images for: The Intrinsic Resolution Limit in the Atomic Force Microscope: Implications for Heights of Nano-Scale Features
Source: PLoS One. 2011 Aug 30;6(8):e23821. doi: 10.1371/journal.pone.0023821 (PMC3166059; doi:10.1371/journal.pone.0023821)

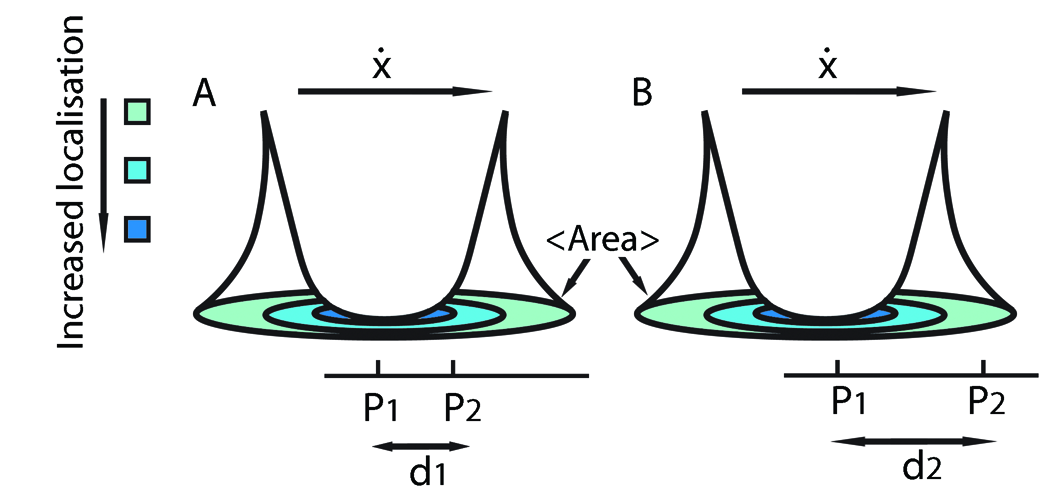

Supplement: Figure S1 — Scheme of the effective interaction area, , of a tip in the proximity of a surface and its relationship to sensitivity and proximity of surface features. The force per unit area might change from the point just under the tip (P1) to other points inside (e.g. P2). This is exemplified with differences in contrast where, in the example, the darker the colour the greater the localisation. For example the force per unit area (or localisation) is larger in (a) d1 than (b) d2 where d2>d1. (TIF) [file pone.0023821.s001.tif]

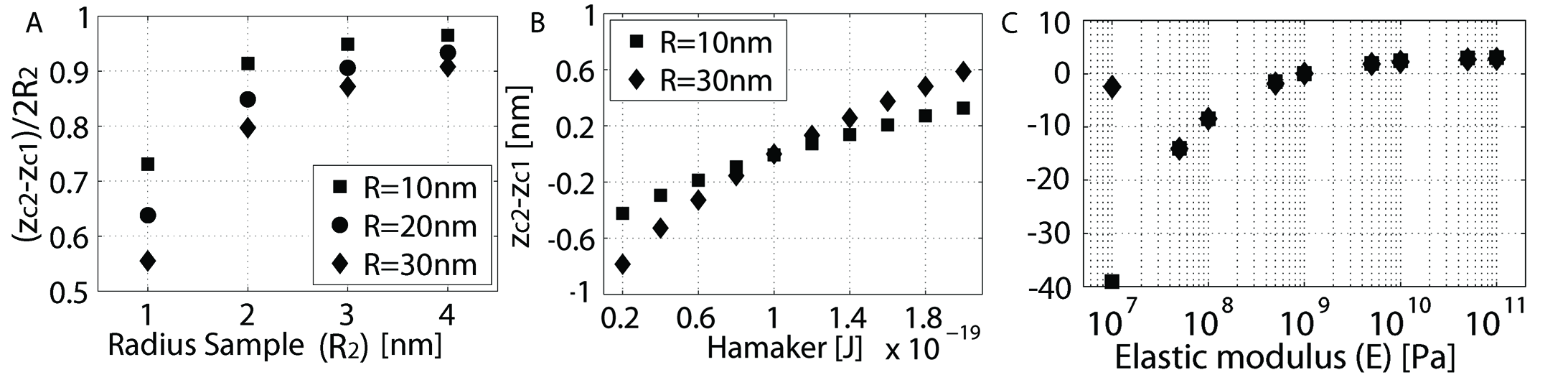

Supplement: Figure S2 — Simulations where the predicted apparent height of surface samples is shown. (a) Changes in apparent height (zc2−zc1) in the non-contact mode for a tip-surface-sample system (spheres, R2 = 1–4 nm) for several values of tip radius (R = 10, 20 and 30 nm). The value zc2−zc1/2R2 decreases with R2 and increases with decreasing R even though the characteristic parameters of both sample (i.e. a sphere) and surface are the same, except for the elastic modulus. However, since all measurements are in the nc mode the value of E is irrelevant here. The parameters are: A0 = 3 m, Es = 0.2 GPa, E = 10 GPa, γ = 30 m (surface and sphere) J/m2, H = 6×10^−20^ J (surface and sphere), Asp/A0 = 0.90. Tip-surface systems. (b) Changes in apparent height (zc2−zc1) due to variations in the local value of H in the nc mode for R = 10 and 30 nm. The parameters are: A0 = 3 m, E = 0.2 GPa, and Asp/A0 = 0.90, γ = 30 m J/m2 (H = 6×10^−20^ J) and the rest as above. The reference value is H = 10×10−20 J (zc1). (c) Changes in apparent height due to variations in the local values of the E in the repulsive regime. Values for R = 10 nm and R = 30 nm overlap in this case because very large free amplitudes were used except for the case E = 10 MPa and R = 30 nm for which the repulsive regime could not be reached. The parameters are: A0 = 60 nm, Asp/A0 = 0.70 and the rest as above. The reference value is E = 1 GPa (zc1). (TIF) [file pone.0023821.s002.tif]

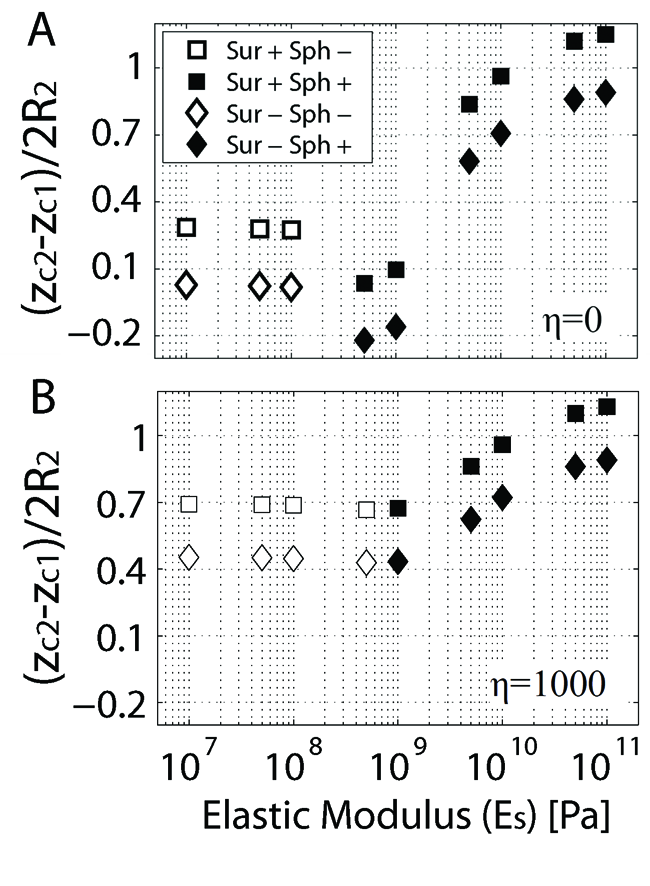

Supplement: Figure S3 — Predictions of apparent height as a function of elastic modulus of the sample including viscoelasticity. (a) As Fig. 4a, i.e. no viscoelasticity. (b) Consequences of including viscoelasticity (η = 1000 Pa•s2). The meaning of the markers is also the same as that in Fig. 4a. This viscoelastic term, previously used in the literature for the typical case of tip-surface only [34], [41], provides a dissipative mechanism in which dissipation increases with indentation. The height difference between squares and rhombuses is a consequence of the attractive regime being reached on the sample sphere in the former and the repulsive in the latter case. (TIF) [file pone.0023821.s003.tif]

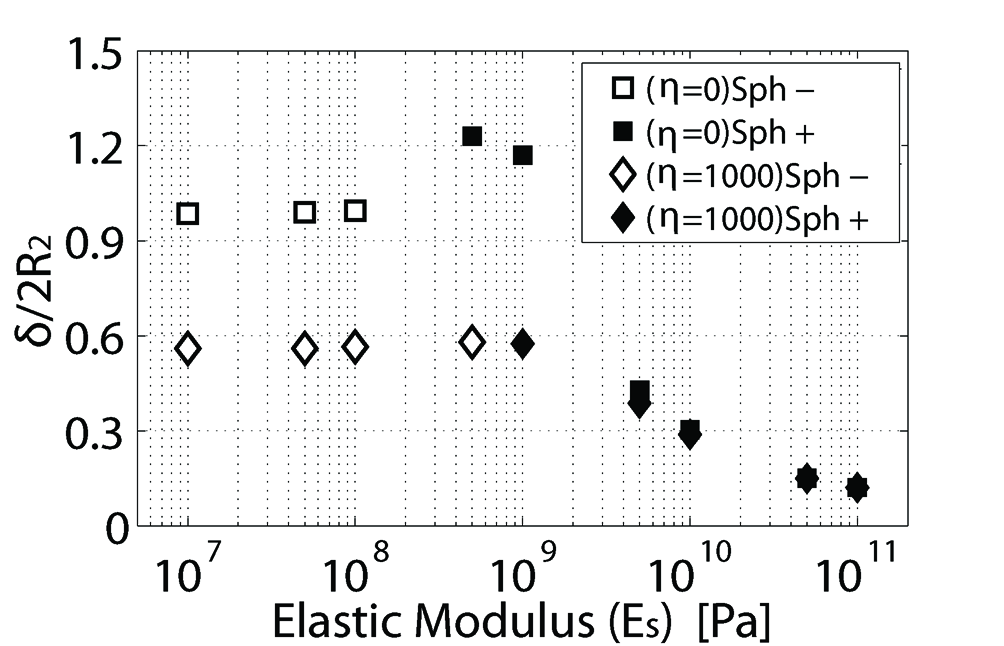

Supplement: Figure S4 — Predictions for the normalised deformation (or indentation) for a sample sphere of R2 = 1 nm. The indentations correspond to those in Fig. 4a and S3. The maximum deformation occurs for intermediately compliant samples (e.g. 0.5<Es<2 GPa) both when no viscoelasticity is allowed (squares) and when it is present (rhombuses); less so in the latter. The legends are different to those in Figs. 4a and S3; S4 only shows whether the force regime is attractive (−) or repulsive (+) on the sphere (Sph). (TIF) [file pone.0023821.s004.tif]

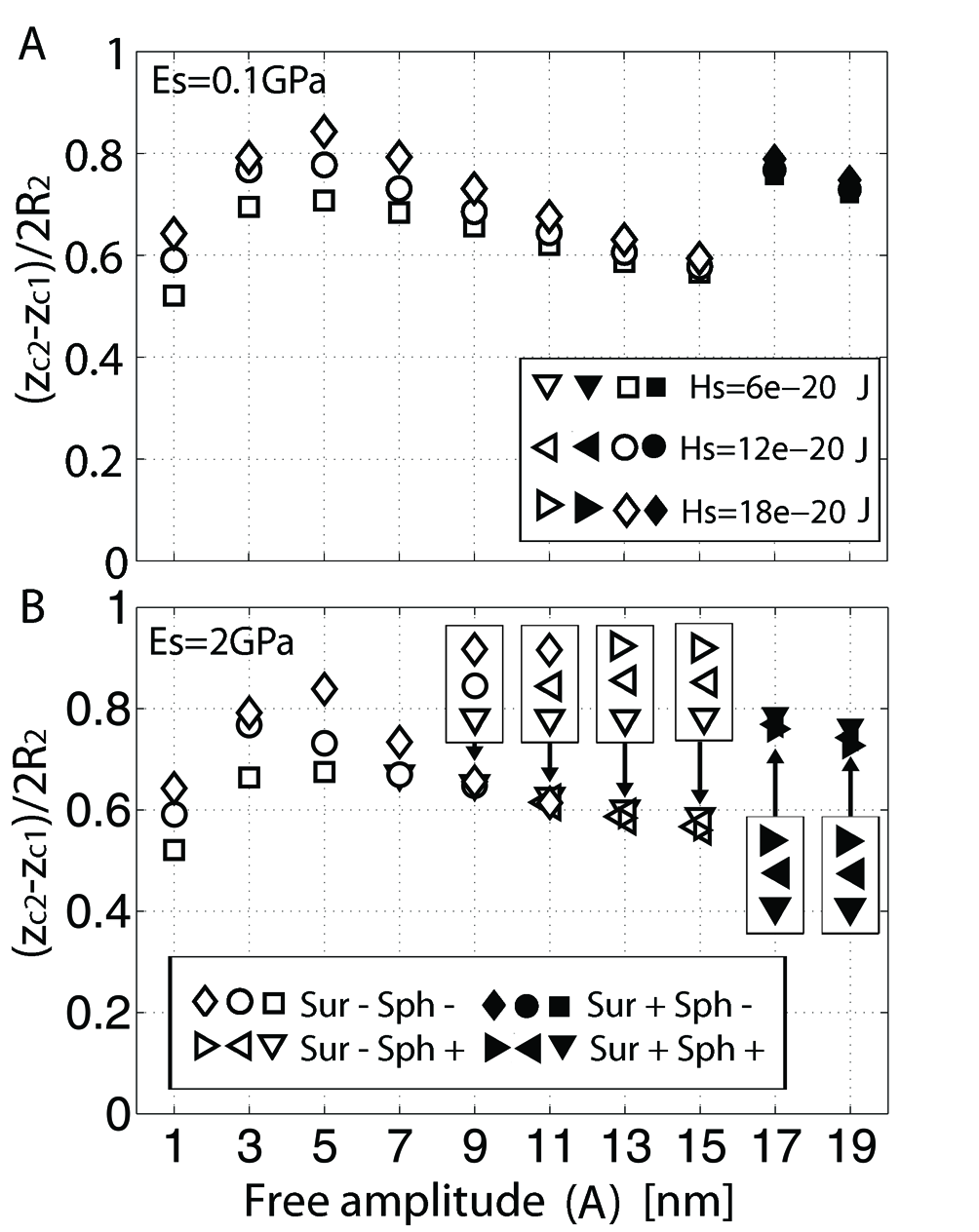

Supplement: Figure S5 — Predictions of apparent height as a function of free amplitude, elastic modulus and Hamaker. Apparent height (zc2−zc1)/2R2 for (a) a compliant sample (Es = 0.1 GPa) and (b) an intermediately compliant sample (Es = 2 GPa) as a function of free amplitude for a constant set-point. Where the markers overlap these are shown explicitly with arrows pointing to the respective points. If no viscoelasticity is allowed total deformation is predicted in some cases (data not shown). The parameters are: Asp/A0 = 0.88, E = 10 GPa, γ = 60 m J/m2, H = 12×10−20 J and η = 1000 Pa•s2. (TIF) [file pone.0023821.s005.tif]

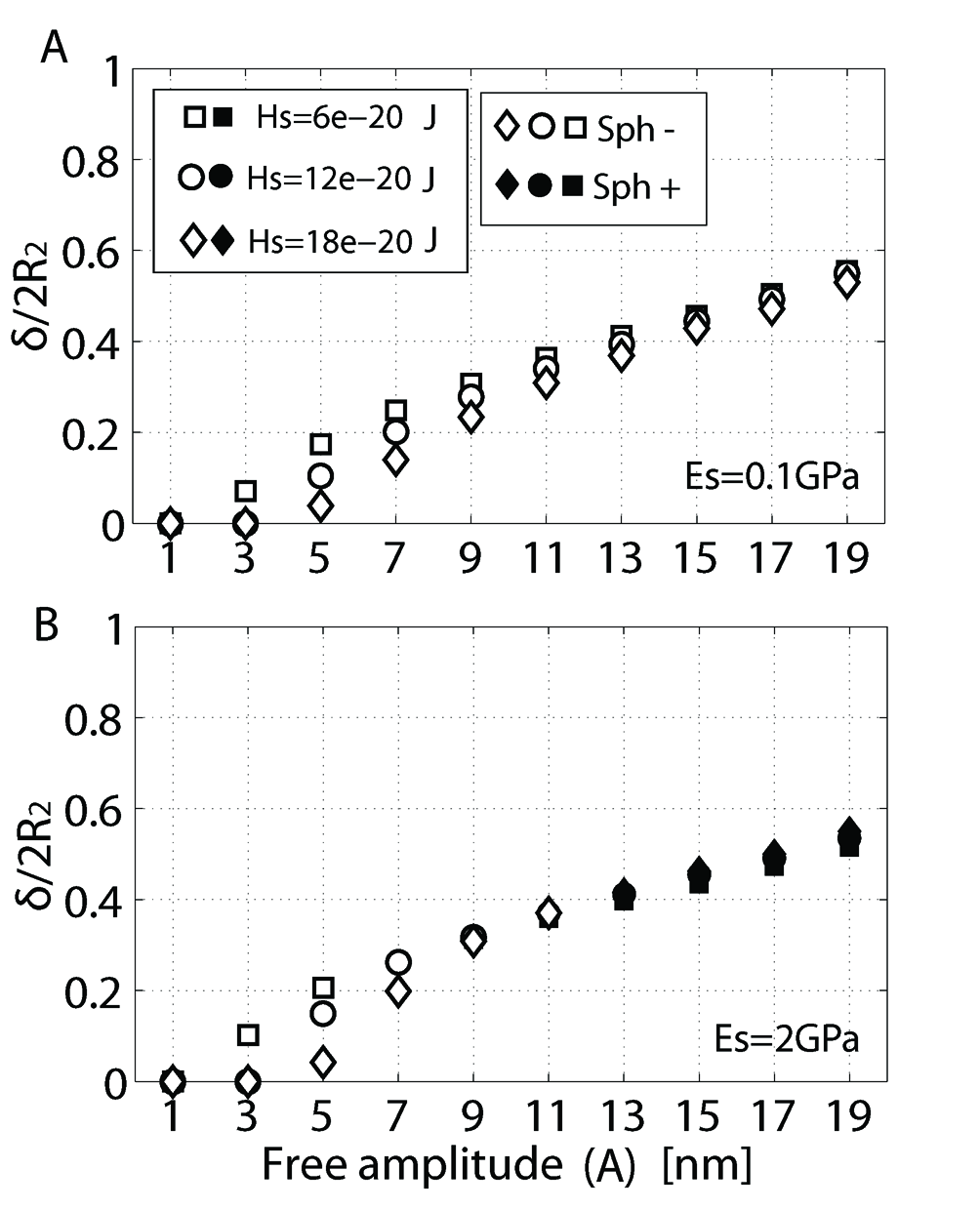

Supplement: Figure S6 — Predictions for the Normalised deformation as a function of A0 corresponding to the same parameters used to obtain Fig. S5. The deformation monotonically increases with A0 but does not correspond to a pattern that might be expected if reduction in apparent height, (zc2−zc1)/2R2 was a consequence of deformation only (c.f. Figs. S5, S6). In particular, it is remarkable that even though there is a step up in (zc2−zc1)/2R2 when the repulsive regime is reached on the surface (A0>15 nm) the deformation still increases. (TIF) [file pone.0023821.s006.tif]

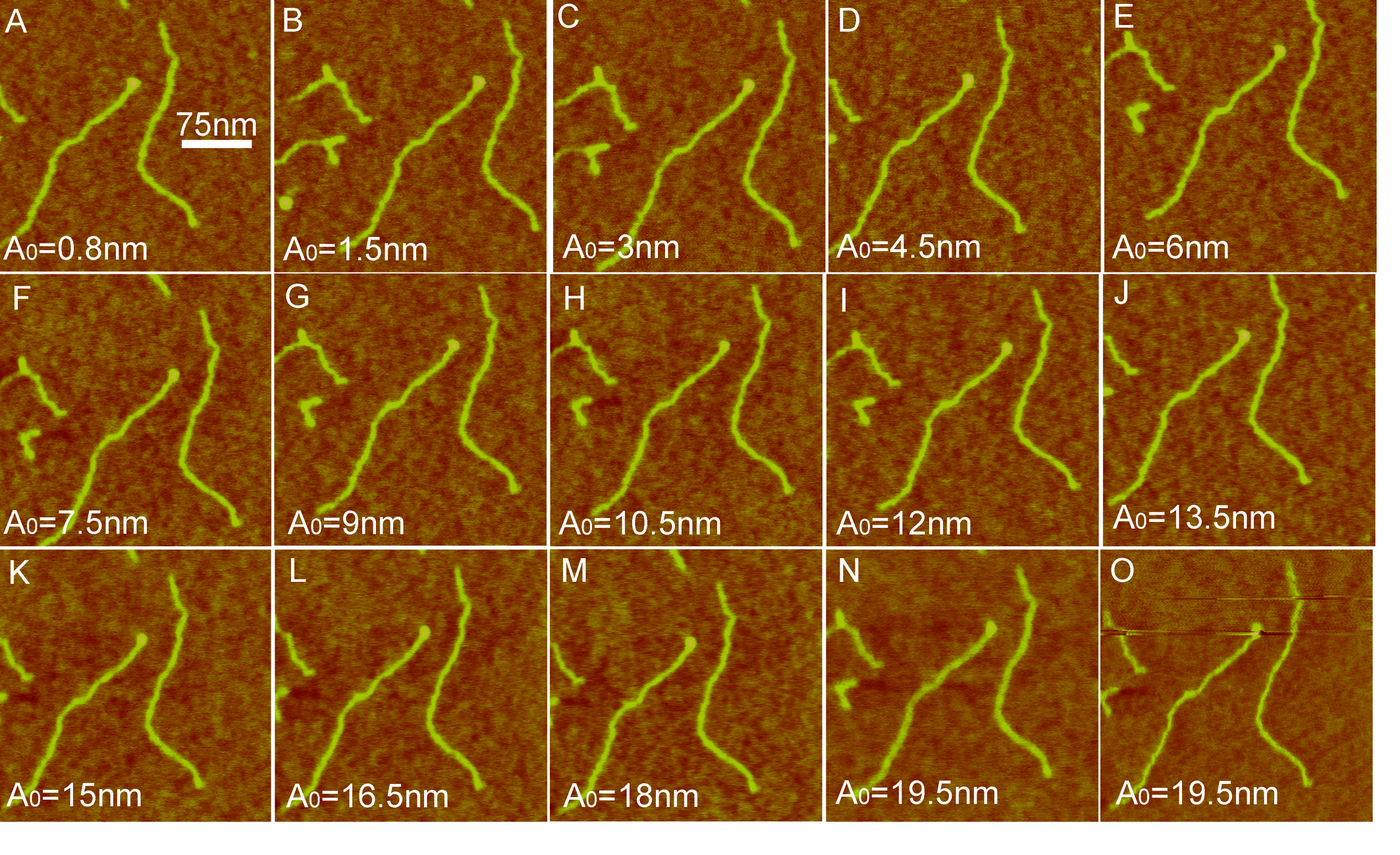

Supplement: Figure S7 — Sequence of topographic scans of two 800 Kbp dsDNA molecules. (a–o) A0 has been systematically increased (1<A0<20 nm) while keeping the set-point high and driving at resonance. All scans have been acquired with Asp/A0∼0.9 except for (o) where Asp/A0 has been slightly reduced compared to (n), in order to allow comparison of apparent heights between the L-state (attractive regime) and H-state (repulsive regime) there. The value of R was approximately 10 nm. (TIF) [file pone.0023821.s007.tif]

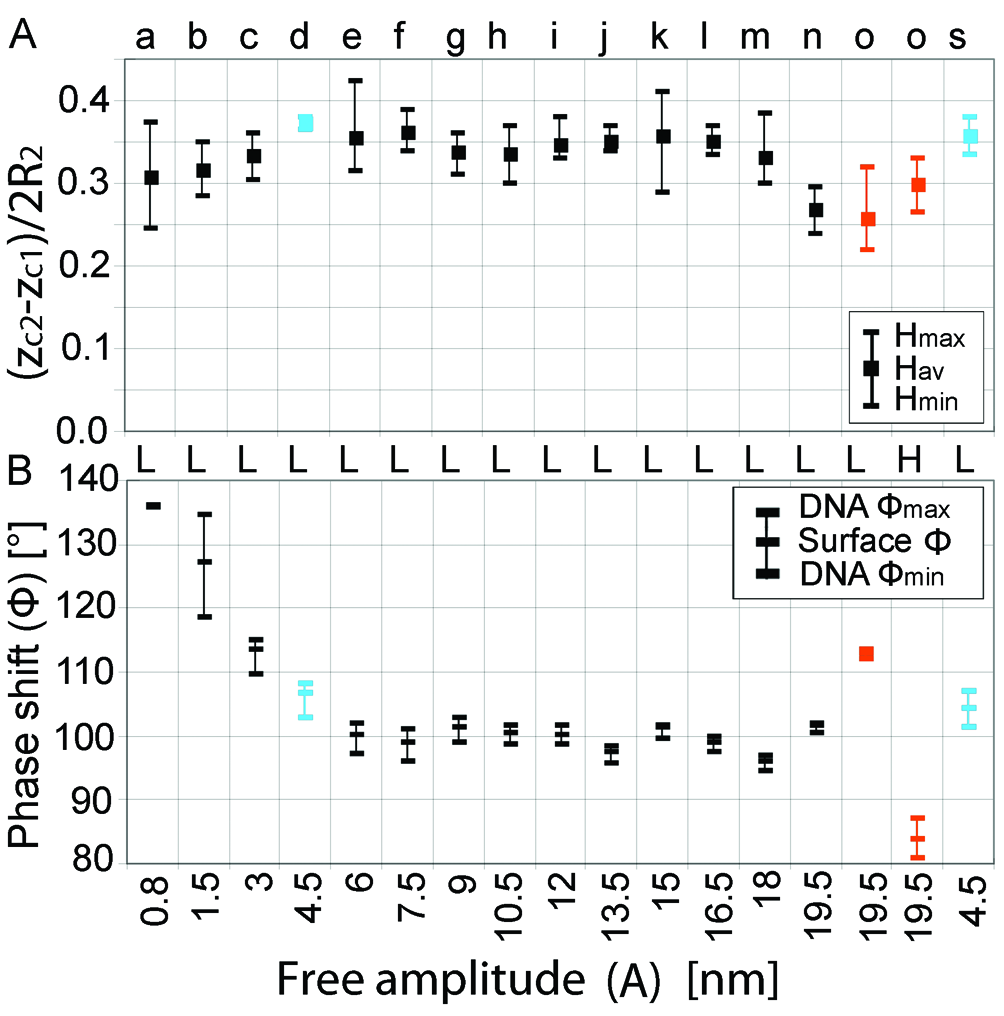

Supplement: Figure S8 — Experimental values of (zc2−zc1)/2R2 and phase shift corresponding to Fig. S7. (a) Experimental values of (zc2−zc1)/2R2, where R2 = 1 nm has been taken as the reference value for the true radius of dsDNA molecules. Even though the DNA molecules are certainly not perfectly circular in cross-section, the predicted values can be taken as a first approximation to the phenomenon. Average values and error scales are shown. Note that an extra value is shown at the end for A0 = 4.5 nm. This corresponds to a control scan obtained to compare the values of (zc2−zc1)/2R2 after the sequence (scan not shown). The two values of A0 = 4.5 nm are coloured blue to allow easy comparison. There are also two values for A0 = 19.5 nm corresponding to the attractive and repulsive regions in Fig. S7o; shown in red. These allow for comparison between regimes for these larger values of A0 and, as predicted (Fig. S5) (zc2−zc1)/2R2 is larger in the repulsive regime! This is despite δ also being larger in the repulsive regime. This outcome is also demonstrated in the main text in Fig. 3. Nevertheless it is important to realise that, in general, these type of simulations predict that for relatively large values of A0, (zc2−zc1)/2R2 can be larger in the repulsive regime for a given Asp/A0. From this, it does not necessarily follow that (zc2−zc1)/2R2 increases with A0. In particular, the tendency for increasing A0 is that (zc2−zc1)/2R2 decreases with increasing A0 once in the repulsive regime and this is confirmed in simulations for A0>19 nm such as that shown in Fig. S5. Experimental evidence of this behaviour can be found in the literature [19]. (b) Corresponding phase shifts where L and H on top of each data point stand for L and H-states respectively. (TIF) [file pone.0023821.s008.tif]

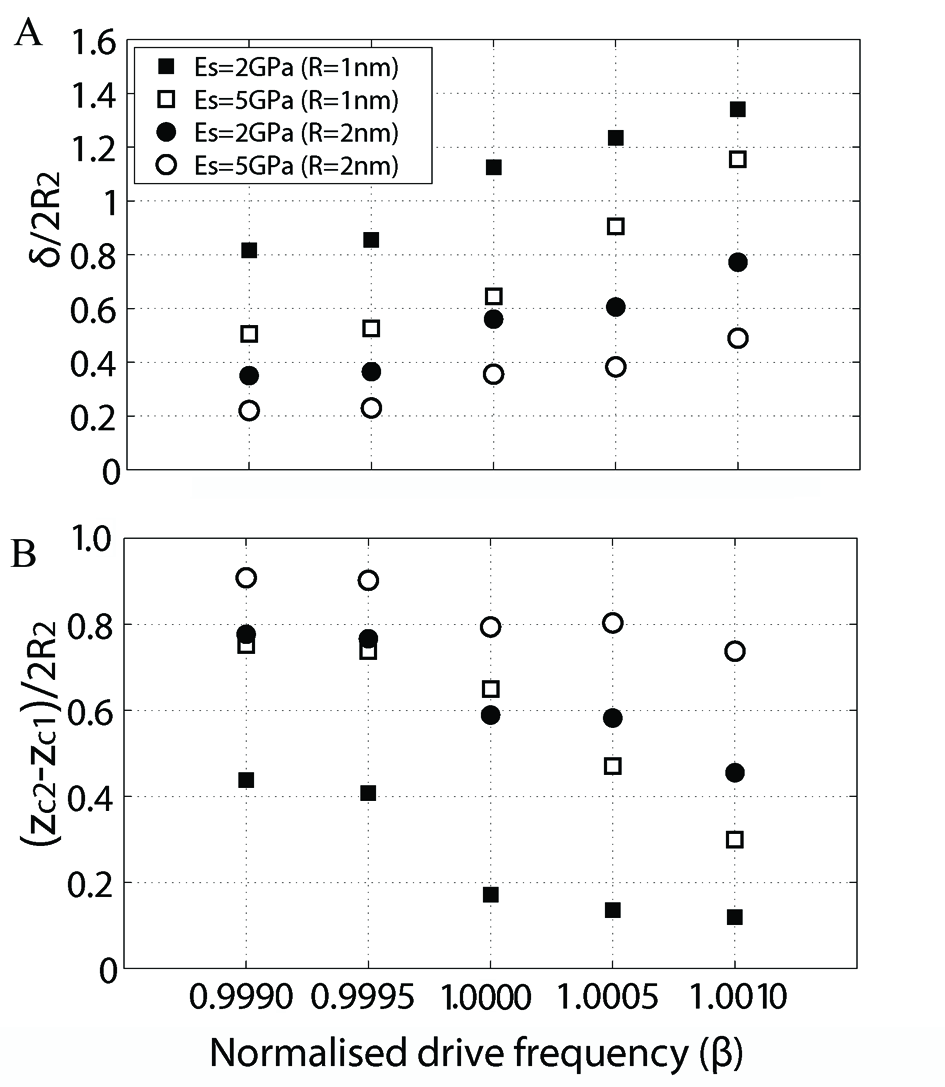

Supplement: Figure S9 — Predictions relative to drive frequency in the repulsive regime. Simulations of (a) Normalized δ and (b) (zc2−zc1)/2R2 for Es = 2 GPa (filled) and 5 GPa (outlined) of R2 = 1 (squares) and 2 nm (circles). A value of A0 = 24 nm and set-point of Asp/A = 0.80 has been used throughout. No viscoelastic term has been used (e.g. η = 0 Pa•s2). Here δ is seen to increase with increasing drive frequency while ((zc2−zc1)/2R2) decreases. All values have been obtained in the repulsive regime. There is a clear relationship between increasing δ and decreasing (zc2−zc1)/2R2. The parameters are: H = 10×10−20 J and R = 10 nm and the rest as indicated in the main text. (TIF) [file pone.0023821.s009.tif]

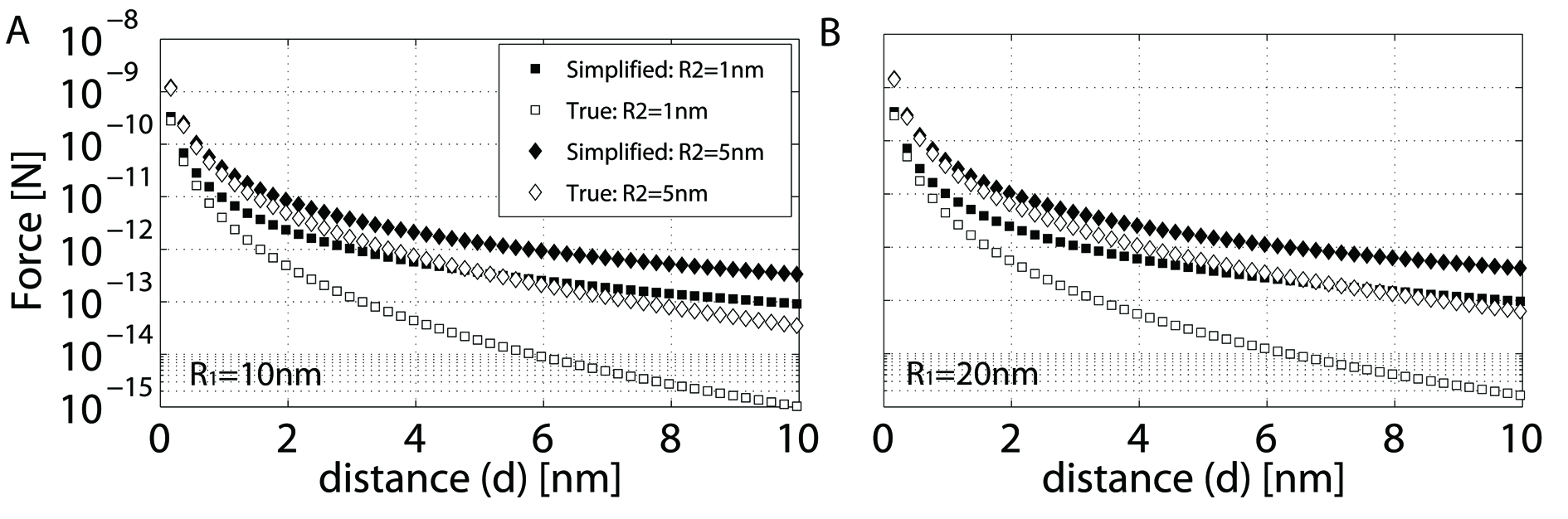

Supplement: Figure S10 — Comparison between exact and simplified van der Waals expressions for two spheres. Force versus distance between a tip (R = R1) and a sphere (R2) for (a) R = 10 nm and (b) 20 nm. The forces predicted by the simplified van der Waals equations are shown (filled) against the true values (outlined) for R2 = 1 (squares) and 5 nm (rhombuses) respectively. (TIF) [file pone.0023821.s010.tif]

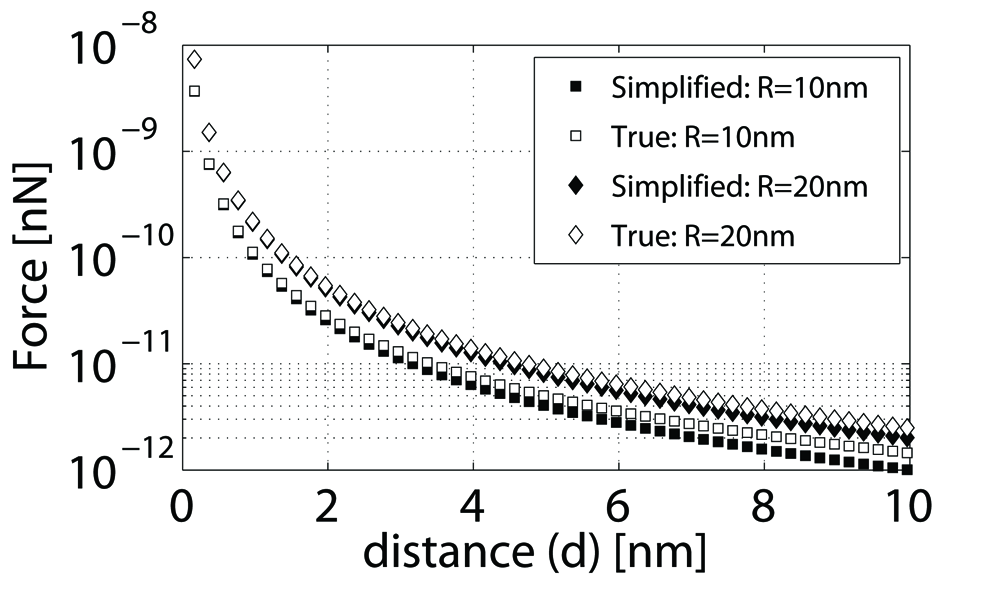

Supplement: Figure S11 — Comparison between exact and simplified van der Waals expressions for an infinite surface and an sphere. Comparison between the simplified equations (filled) for the van der Waals force between a sphere of radius R and an infinite and flat surface and the true equations (outlined). The forces are shown as a function of distance d. The relationships are shown for R = 10 (squares) and 20 nm (rhombuses). The simplified form closely follows the true equation even for distances as large as 3 and 4 nm. (TIF) [file pone.0023821.s011.tif]

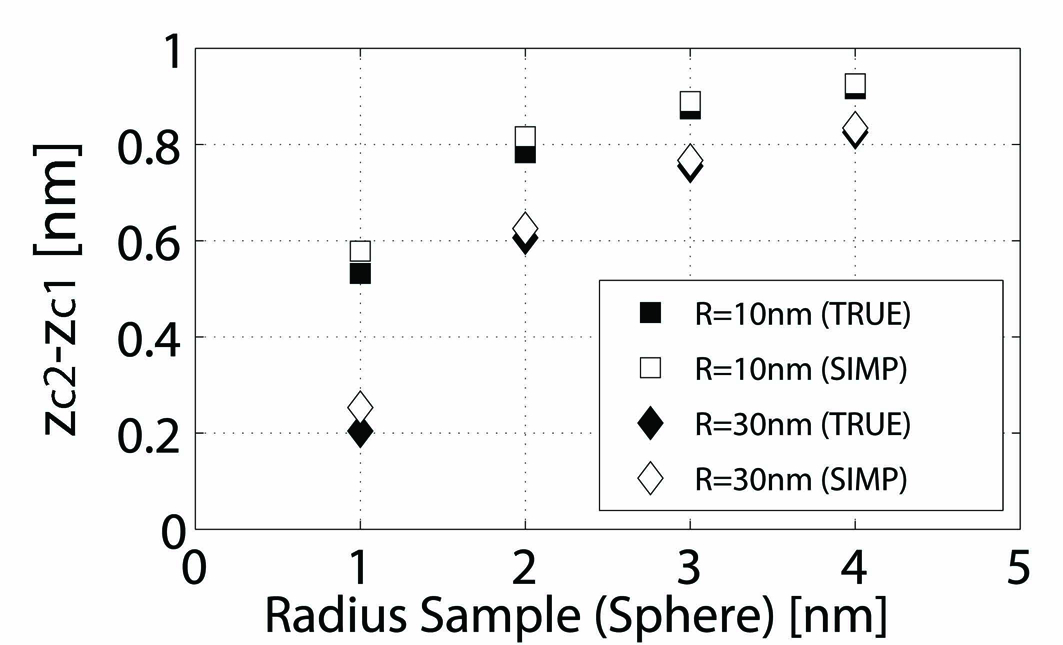

Supplement: Figure S12 — Comparison between the results for apparent height between the exact and simplified van der Waals expressions in the nc mode. Simulations showing (zc2−zc1)/2R2 for several values of R and R2. The true equations for the van der Waals interactions (filled) have been used against the simplified equations (outlined) for R = 10 and 30 nm respectively. The true values produce only slightly smaller values for the apparent height. All data was achieved in the non contact mode. The parameters are: A0 = 1 nm, Asp/A0 = 0.95, Es = 5 GPa, E = 10 GPa, H = 6.1×10^−20^ J, γ = 30 m J/m2 and all other parameters as detailed in the main article. (TIF) [file pone.0023821.s012.tif]
